# Transcriptome sequencing analysis of echovirus 30 infection reveals its potential pathogenesis

**DOI:** 10.3389/fmicb.2022.958385

**Published:** 2022-09-06

**Authors:** Qiang Sun, Jichen Li, Bo Zhang, Rui Wang, Congcong Wang, Xiaoliang Li, Ying Liu, Yong Zhang

**Affiliations:** ^1^WHO WPRO Regional Polio Reference Laboratory, National Health Commission Key Laboratory for Biosafety, National Health Commission Key Laboratory for Medical Virology, National Institute for Viral Disease Control and Prevention, Chinese Center for Disease Control and Prevention, Beijing, China; ^2^Teaching Department of Basic Medicine, Taishan Vocational College of Nursing, Tai’a, China; ^3^Center for Biosafety Mega-Science, Chinese Academy of Sciences, Wuhan, China

**Keywords:** echovirus 30, transcriptome sequencing, human glioma cell, mouse brain tissue, differentially expressed genes

## Abstract

Echovirus 30 (E30) causes various diseases, such as viral encephalitis; aseptic meningitis; hand, foot, and mouth diseases; and acute flaccid paralysis. Related neurological infections are most concerning. However, the molecular mechanisms of E30 pathogenesis are not fully understood. There is a growing research interest in E30 as a cause of neurological disease. The aim of this study was to describe E30 infection, especially the changes in differential factor expressions after infection, in human glioma (U251) cells and mice brains using transcriptome sequencing analysis. Clear changes in the gene expression of factors associated with the defense response to viruses, inflammation-related signaling pathways, and neurological complication-related pathways were observed. Our results suggest that after E30 infection, the genes related to immune response were induced in the human glioma cells and mice brains, whereas genes functioning in the development and function of neural tissue were inhibited. Overall, this study successfully established E30 infection of U251 and mouse brain tissue, profiled the infection-induced changes in cellular and organizational transcriptomes, and revealed the molecular level changes during E30 infection.

## Introduction

Enteroviruses (EVs) are commonly found worldwide and cause a variety of diseases, such as viral encephalitis; aseptic meningitis; hand, foot, and mouth diseases; and acute flaccid paralysis (Knowles et al., 2012). EVs belonging to the order *Picornavirales* of the *Picornaviridea* family are non-enveloped viruses and have a positive single-stranded RNA genome (Khetsuriani et al., 2006). Echovirus 30 (E30) belongs to the Enterovirus B genus and is one of the most frequently detected enterovirus; it is often detected in patients with neurological infections (McWilliam Leitch et al., 2009; Chen et al., 2020; Benschop et al., 2021). Although E30 is a dangerous human pathogen, no vaccine or effective drugs are present for it. The life cycle of E30, similar to other EVs, depends entirely on the host cell system. However, the exact mechanism of damage caused by E30 infection is not clear. Therefore, it is essential to explore the mechanism of how host-cell interaction affects the viral replication and pathogenesis.

RNA sequencing (RNA-Seq) is widely used to investigate the differences in gene expressions at genome-wide levels (Stark et al., 2019; Zhang et al., 2021). Many studies have analyzed the transcriptome expression profiles of microbial infections, which provide clues regarding pathogenic mechanisms (Poma et al., 2020; Song et al., 2020; Li et al., 2021). At present, most of the data associated with RNA-Seq of EVs are about enterovirus 71 (EV-A71) and coxsackievirus A16 (CVA16) (Song et al., 2020). In contrast, RNA-Seq data about E30, which is a virulent EV, are lacking, and hence, there is an urgent need to further supplement the RNA-Seq analysis.

Several studies have indicated that EV-A71 is a neurophagic virus that preferentially infects astrocytes in the brains of mice and primate animal models; they demonstrated that astrocytes play a key role in the viral neurogenic pathogenesis (Ruller et al., 2012; Lin and Shih, 2014). Moreover, E30 infections frequently lead to severe central nervous system (CNS) complications and even death. In this study, we aimed to reveal E30-triggered changes at the neural-related cell and tissue level by analyzing the transcriptome of human glioma (U251) cells and mouse brain after E30 infection.

Compared with the previous studies, besides the inflammation, interferon (IFN) signaling, and immune-related gene changes, we observed neurological complication-related gene changes. Overall, the aim of this study was to reveal extensive transcriptional responses that could provide important, new data to supplement and perfect the E30 pathogenic mechanism.

## Materials and methods

### Cells and virus

Human glioma (U251, ATCC) cells were cultivated in Dulbecco’s modified Eagle’s medium (DMEM) and F-12 (Gibco, United States) at 37°C with 5% CO_2_. The E30 strain, TL1C/NM/CHN/2016, isolated from an outbreak of aseptic meningitis in Tongliao city, Inner Mongolia Autonomous Region, China, from June to August 2016, was used for infection [multiplicity of infection (MOI) = 0.01]. E30-infected U251 cells and control U251 cells were collected at 48-h after infection for transcriptome sequencing analysis.

### *In vivo* study

A previous study (PMID: 35420443) has demonstrated that no effective replication of E30 (strain WZ16) was found in the brain and other organs of wild-type C57 suckling mice by intracranial injecting E30 (Li et al., 2022). To better understand the effect of E30 on other types of mice, we selected E30 (strain TL1C/NM/CHN/2016) to infect Institute of Cancer Research (ICR) suckling mice, which are one of the most sensitive animals to human enterovirus. ICR mice were purchased from SPF Biotechnology Co., Ltd, (Beijing, China). Suckling mice were infected with 50 μl 10^6^TCID_50_ E30 or culture medium [DEME/F12 basic and 2% fetal bovine serum (FBS)] *via* intramuscular injection. Weight, survival rate, and clinical scores of mice were recorded daily. The clinical disease standards were scored as follows: 0, no disease; 1, ruffled fur; 2, weight loss; 3, single limb paralysis; 4, both hind limb paralysis; and 5, moribund or dead. Mock-infected suckling mice in the E30-infected group were sacrificed 5 days after infection for brain tissue collection and transcriptome sequencing analysis.

### Viral titer in U251 cell and mouse brain tissue

After all samples are ready, detailed viral titer determination assay procedures are as follows: eleven 10-fold dilution gradients were performed for each sample to be tested, with 10^–1^ to 10^–11^ dilution, and three parallel wells were performed for each dilution. A volume of 100 μl diluted sample was added to wells in columns 1–11 of a 96-well plate and column 12 of the cell control. Then, 100 μl of cell suspension containing 2 × 10^5^ cells/ml was added to all wells. The plates were then incubated at 36°C in a CO_2_ incubator. Cytopathic effect (CPE) reading was performed daily using an inverted microscope and was recorded for 5 days. For a valid test, the cell control should have a complete monolayer of healthy cells. Viral titers were finally calculated by the Kärber formula.

### RNA extraction and quantification

Total RNA was extracted from the samples of E30-infected and control groups (each containing three samples) in U251 cells and mouse brain tissues by TRIzol reagent (Invitrogen, United States). The RNA amount and integrity were assessed using the RNA Nano 6000 Assay Kit of the Bioanalyzer 2100 system (Agilent, United States). A total of 12 samples were independently subjected to RNA-Seq library construction.

### Library preparation for transcriptome sequencing

mRNA was purified from total RNA using polyA, oligosaccharide-linked magnetic beads (New England BioLabs, United States). The fragments were then broken down into smaller fragments and the first cDNA strand was synthesized using M-MuLV reverse transcriptase. The second cDNA strand was treated with dNTPs. DNA fragments were adenylated at the 3′ end and ligated with sequencing adapters. Fragments (370–420 bp in size) were polymerase chain reaction (PCR) amplified using Phusion High-Fidelity DNA Polymerase (Thermo Scientific, United States), and the PCR product was purified using AMPure XP beads and the library was finally obtained.

### Clustering and sequencing

The Illumina NovaSeq 6000 was used to sequence different libraries according to the requirements of effective concentration (higher than 2 nM) and target offline data volume (6G bases), and 150 bp paired-end readings were generated. The sequencer captured the fluorescence signal and converted the optical signal into a sequencing peak using in-house computer software to obtain the sequence information of the fragment to be tested.

### Data analysis

#### Quality control

The image data measured using the high-throughput sequencer were converted into sequence data (reads) using CASAVA base recognition. Raw data (raw reads) in fastq format were first processed using the Fast QC software package. At the same time, the Q20 (represents an error rate of 1%, general Q20 > 95% is considered qualified), Q30 (represents an error rate of 0.1%, general Q30 > 80% is considered qualified), and GC contents (sampling statistics percentage of GC content in reads sequences, GC content is between 40 and 60%, and quality is acceptable) of the clean data were calculated.

#### Mapping reads to the reference genome

The index of the reference genome was built using HISAT2 (version 2.0.5) and paired-end clean reads were aligned to the reference genome using HISAT2 (version 2.0.5) (Kim et al., 2019). HISAT2 can generate spliced, linked databases based on gene model annotation files, thus providing better comparison results than other non-spliced comparison tools.

#### Quantification of gene expression level

Feature counts (version 1.5.0-p3) were used to count the read numbers mapped to each gene. Then, the FPKM of each gene was calculated based on the length of the gene and the read count mapped to this gene. FPKM refers to the expected number of fragments per kilobase per million base pairs of the sequenced transcripts.

#### Differential expression analysis

Differential expression analysis of the two conditions/groups (three biological replicates per condition) was performed using the DESeq2 package in R (version 1.20.0) (Liu et al., 2021). DESeq2 provides statistical routines for determining differential expression in digital gene expression data using a model based on a negative binomial distribution. The resulting *P*-values were adjusted using Benjamini and Hochberg’s approach to control the false discovery rate. Padj ≤ 0.05 and | log2 (foldchange)| ≥ 1 were set as the thresholds for significantly differential expression. Principal component analysis (PCA) is also commonly used to evaluate the differences between groups and between different samples within groups. PCA adopts the calculation method of linear algebra to carry out dimension reduction and principal component extraction for tens of thousands of genetic variables. In this study, all samples were analyzed by PCA based on gene expression value (FPKM) using PCA.

#### Gene ontology and kyoto encyclopedia of genes and genomes analysis of differentially expressed genes

The DESeq2 software was used to conduct differential gene expression analysis of the data, and the differential gene expression obtained after the screening was analyzed on the DAVID website.^1^ Gene Ontology (GO) and Kyoto Encyclopedia of Genes and Genomes (KEGG) pathway enrichment analysis databases were interrogated. GO analysis included biological processes (BPs), molecular functions (MFs), and cellular components (CCs). Interactions between representative differentially expressed genes (DEGs) were analyzed using the Search Tool for the Retrieval of Interaction Gene/Proteins (STRING) (Szklarczyk et al., 2021).

#### Validation of differentially expressed genes *via* qRT-PCR assays

Total RNA was extracted from infected mouse brain tissues and reverse transcribed into cDNA. The reaction system was configured and detected on a fluorescence quantitative PCR instrument. Details are given as follows: reverse transcription was performed using a total of 2 μg RNA isolated with PrimeScript II 1st Strand cDNA Synthesis Kit (Takara, Japan) according to the manufacturer’s protocol. Next, qRT-PCR was carried out using 2 μl of cDNA template, 0.75 μl of gene-specific primers, 12.5 μl of SYBR Premix Ex Taq (2 ×), and water up to 25 μl with TB Green Premix Dimer Eraser kit (Takara, Japan). The reaction program was performed as follows: pre-denaturation at 95°C for 30 s, followed by 40 cycles of denaturation at 95°C for 10 s, annealing at 55°C for 15 s, and extension at 72°C for 30 s on a 7,500 Fast Real-Time PCR System (Applied Biosystems, United States). The primer sequences are listed in Table 1. The expression level of related genes was analyzed by the 2^–ΔΔCt^ method, and the β-actin gene was used as an internal reference to compare the differential expression between the selected genes and the control group. All the experimental samples were repeated in three independent experiments.

**TABLE 1:** Primer sequences used for qRT-PCR assays to validate differentially expressed genes.

Primer name	Sequence (5′–3′)
ISG15-F	TGGACAAATGCGACGAACC
ISG15-R	TTCGTCGTTCACTCGCC
MX1-F	GATCATGTGCTGAATGCCTAGCAC
MX1-R	GTGGAGCCTGACCTTGTGGCACTG
GBP2-F	TGGAGGAAGTTTTAGGACGT
GBP2-R	CTCCTCTCTTTTCTTCCGAA
DDX58-F	AGACCCTGGACCCTACCTAC
DDX58-R	GCATCCAAAAAGCCACGGAA
NLRC5-F	ACAGCATCCTTAGACACTCCG
NLRC5-R	CCTTCCCCAAAAGCACGGT
OLIG2-F	TGGGGGCTTGACAAAAGAAC
OLIG2-R	AACAAAGAGCTTCGCATCGC
GPC2-F	CGGAGCCCCTGCACTTC
GPC2-R	GGGCTCACATTCACCTCCTTTA
CCND1-F	GATTGGAGGCACACGTCTCA
CCND1-R	GCTCAGCTACGTTGGTCACT
E2F2-F	AGCTGGATCTGGAGGGGATT
E2F2-R	AGGACCCCATCCTCTGACTC
β-Actin-F	AGCGGGAAATCGTGCGTGAC
β-Actin-R	ATGCCCAGGAAGGAAGGTTG

## Results

### Overview of the transcriptome sequencing

**FIGURE 1:**
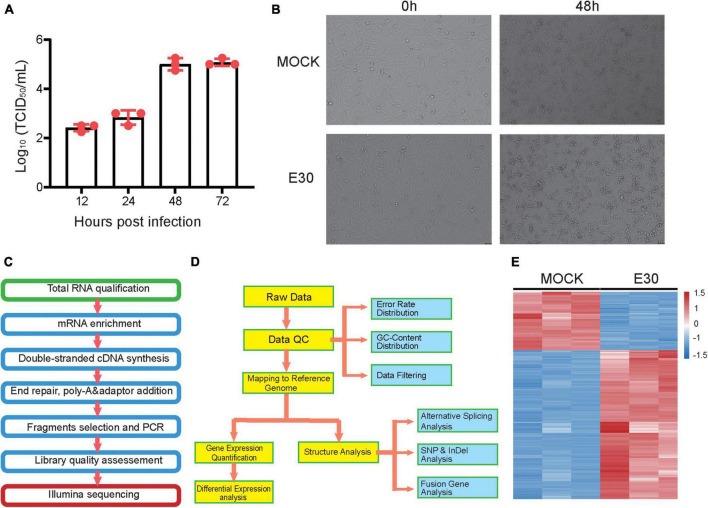
Expression profile for echovirus 30 infection in human glioma (U251) cells. **(A)** Change in E30 viral titer in U251 cells, **(B)** CPE appeared at 48 h after infection of U251 cells with E30, **(C,D)** sequencing and information analysis flow chart, and **(E)** heat map analysis of E30-infected and control group (each group, *n* = 3).

Transcriptome sequencing based on the Illumina sequencing platform is used to study all mRNA transcribed from specific tissues or cells. It is also the basis of gene function and structure research and plays an important role in understanding the development of organisms and the occurrence of diseases. In this study, transcriptome sequencing was used to analyze the changes in differential factor expression after E30 infection of U251 cells. We first determined the replication activity of E30 in U251 cells based on changes in viral titers (Figure 1A). The CPE of U251 cells was also observed at different time points after E30 infection of R U251 cells. Our results showed that E30 replicated well in U251 cells, arriving at a replication plateau 48 h after infection. In addition, 48 h after infection with E30 (MOI = 0.01), U251 cells developed CPE (Figure 1B). Therefore, we selected cell pellets at 48 h after infection for transcriptome sequencing. Briefly, cells were harvested for total RNA extraction and processed for sequencing. Data quality was verified based on the error rate, GC content, and data filtering, and differential gene analysis was performed (Figures 1C,D). We obtained 432,075,80–478,153,48 reads (E30-U251 group) and 434,761,86–477,400,34 reads (control group). A total of 402,559,30–431,196,08 and 402,388,24–444,149,96 clean reads were obtained in the E30-U251 and control groups, respectively. The overall sequencing error rate of the data was 0.03, and the GC percentage of clean reads was 49.5–51.1%, which indicated the reliability of the data (Table 2). A total of 3,273 genes were shown by the heat map to be significantly differentially expressed after E30 infection (Figure 1E).

**TABLE 2:** Summary of the echovirus 30 infected human glioma (U251) cells sequencing data quality.

Sample	Raw_reads	Clean_reads	Error_rate	Q20	Q30	GC_pct
E30-U251	43,207,580	40,255,930	0.03	97.59	93.44	50.29
E30-U251	45,305,842	42,583,728	0.03	97.57	93.46	50.87
E30-U251	47,815,348	43,119,608	0.03	97.75	93.77	49.5
Control-1	47,740,034	44,414,996	0.03	97.34	92.96	51.1
Control-2	43,476,186	40,238,824	0.03	97.4	93.12	50.96
Control-3	47,062,076	43,466,668	0.03	97.37	93.08	51.13

### Gene ontology and kyoto encyclopedia of genes and genomes enrichment analysis of differentially expressed genes in E30-infected U251 cells

**FIGURE 2:**
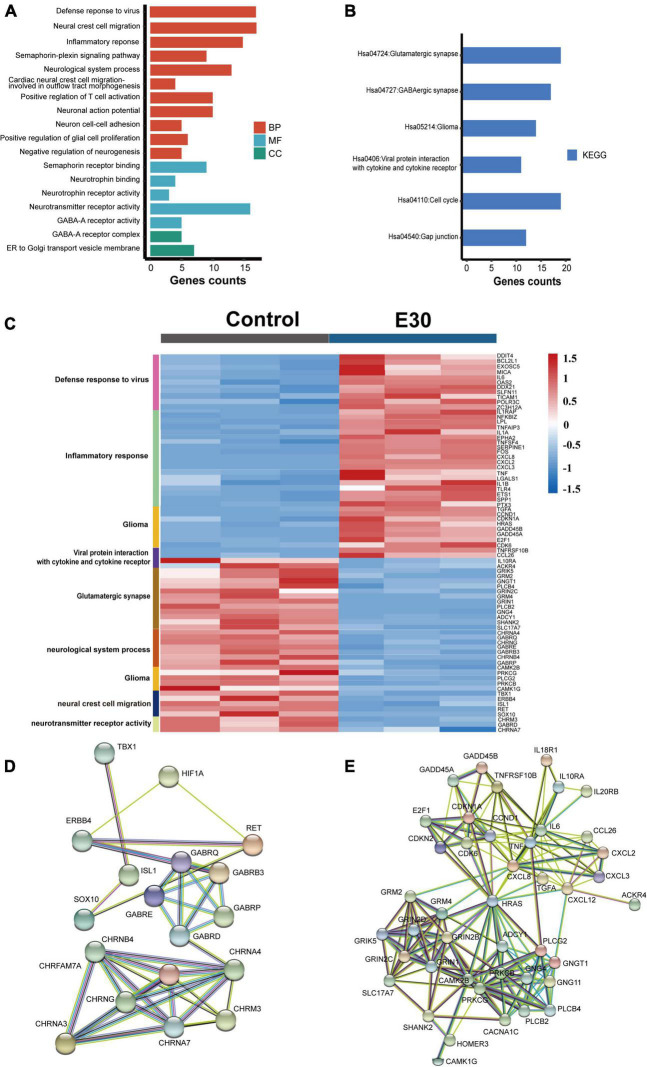
Functional classification of differentially expressed genes (DEGs) in E30-infected human glioma (U251) cells. **(A)** Gene Ontology (GO) enrichment analysis of DEGs. **(B)** Kyoto Encyclopedia of Genes and Genomes (KEGG) pathway analysis of DEGs. **(C)** Visualization of DEGs enriched in E30-infected U251 cells. **(D,E)** Molecular interaction between DEGs and STRING randomly draws colors based on the scores of interacting genes and is labeled with gene names.

Transcriptome analysis provides a better understanding of the viral replication cycle, cellular metabolism, transcription, and other processes. To further explore the transcriptome changes caused by E30 infection of U251 cells, these DEGs were used for GO and KEGG analyses (Supplementary Table 1). Regarding the GO BP terms, E30 induced defense response to virus, inflammatory response, positive regulation of the neural crest cell migration, neurological system process, semaphoring-plexin signaling pathway, neuronal action potential, neuron cell–cell adhesion, positive regulation of glial cell proliferation, T-cell activation, and negative regulation of neurogenesis. DEGs were enriched in five MF and two CC terms, including semaphorin receptor binding, neurotrophin binding, neurotrophin receptor activity, neurotransmitter receptor activity, GABA-A receptor activity, GABA-A receptor complex, and ER to Golgi transport vesicle membranes (Figure 2A). In addition, KEGG pathway analysis of DEGs showed that the enriched terms were the glutamatergic synapse, GABAergic synapse, glioma, viral protein interaction with cytokine and cytokine receptors, cell cycle, and gap junction (Figure 2B). These data suggest that E30 infection is associated with the innate immune response to viral infection and genetic neurogenic dysregulation. Through further visualization analysis of differential genes, we found that some genes associated with inflammatory response (ZC3H12A, POLR3C, TL1RAP, NFKBIZ, CXCL2, and CXCL8) and defense response to viruses (DDIT4, BCL2L1, EXOSC5, MICA, IL6, OAS2, and DDX21) were significantly upregulated, while some genes associated with neuropathy (GRIK5, GRM2, GNGT1, CHRNG, PRKCG2, GABRD, GRIN1, GABRP, TBX1, and SOX10) were downregulated (Figure 2C). Finally, the STRING network was used to identify the molecular interactions of these DEGs in neuropathy-related pathways and inflammatory responses. Specifically, these DEGs were associated with neuropathy-related pathways, such as GABRD, GABRP, TBX1, and SOX10 (Figure 2D), and inflammatory responses such as CXCL3, CXCL12, TNF, and IL6 (Figure 2E).

### Transcriptome analysis of E30-infected institute of cancer research suckling mouse brain tissue

**FIGURE 3:**
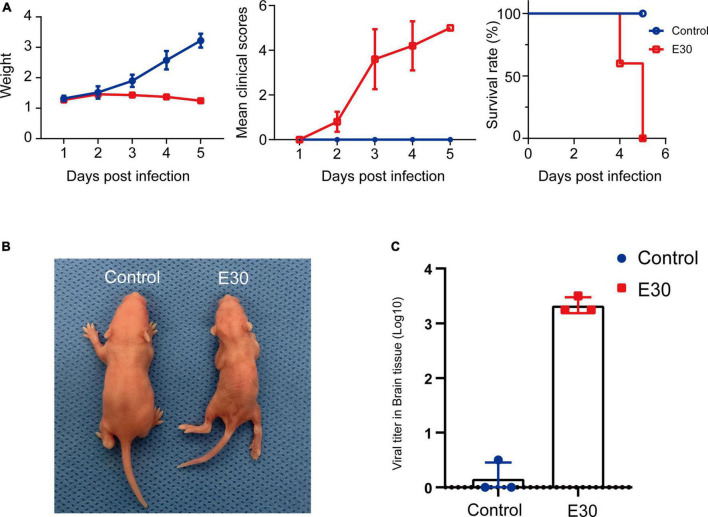
E30 infects ICR suckling mice. **(A)** Body weight, clinical score, and survival in ICR suckling mice infected with E30. **(B)** Clinical symptoms of E30 infection in ICR suckling mice. **(C)** Virus tissues in brain tissues 5 days after infection.

**FIGURE 4:**
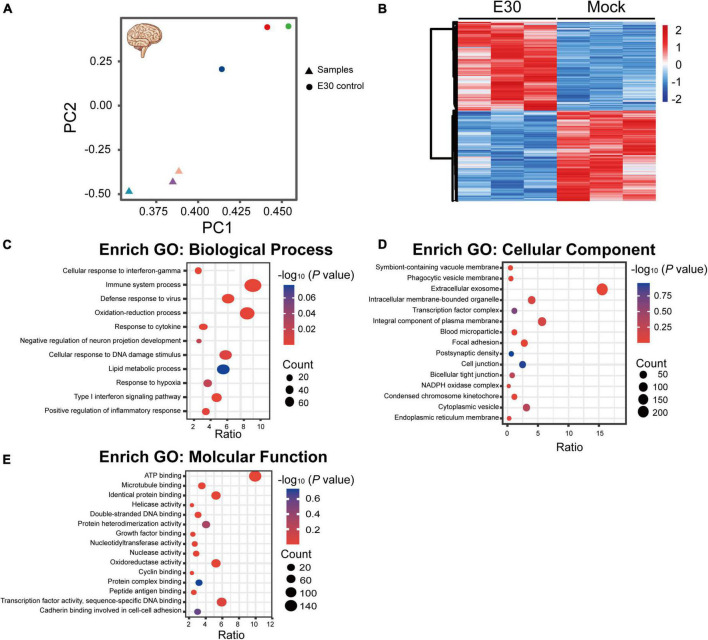
Functional classification of differentially expressed genes (DEGs) in E30-infected suckling mice brain tissue. **(A,B)** Principal component analysis (PCA) and heat map analysis of E30-infected brain tissue and control infection group (each group, *n* = 3), **(C)** enriched pathways among DEGs in biological processes, **(D)** cellular components, and **(E)** molecular function terms. High *P*-values are in blue and low *P*-values are in red. The size of the spots indicates the number of genes.

After intramuscular injection of E30 (strain TL1C/NM/CHN/2016) into ICR suckling mice, on days 1–4 after E30 infection, the average body weight of mice in the E30-infected group slowly increased, and the daily average body weight was lower than that of the control group (94.07–64.36%), and on day 5, the average body weight of mice in the E30-infected group decreased significantly, and the average body weight of mice in the E30-infected group was only 46.58% than that of mice in the control group, showing a significant weight loss. Compared with the control group, the E30-infected group presented with clinical manifestations of hind limb muscle paralysis 3 days after infection and even died on day 4 (Figures 3A,B). Although some mice started to die at 4 days after infection, to ensure the consistency of the experiment, mock-infected suckling mice in the E30-infected group were sacrificed at 5 days after infection for brain tissue collection, virus titer determination, and transcriptome sequencing analysis. Titers in brain tissues were reaching 10^3⋅5^ TCID_50_ at 5 days after infection (Figure 3C). After removing the low-quality reads from the original data, 423,921,50–504,301,26 (E30-brain group) and 457,932,04–477,426,74 (control group) clean reads were obtained. The overall sequencing error rate of the data was 0.02, and the GC percentage of clean reads was 48.13–49.68%, which indicated the reliability of the data (Table 3). PCA was used to evaluate the degree of dispersion between the two groups, and a heat map was used to analyze the data differences between each group. E30 infection produced different results from the control group (Figures 4A,B). The results of GO BP terms showed that the DEGs were enriched in the type I IFN signaling pathway, immune system process, defense response to virus, and oxidation-reduction process; CC and MF terms included symbiont-containing vacuole membrane, phagocytic vesicle membrane, extracellular exosome, focal adhesion, cell junction, ATP binding, microtubule binding, helicase activity, and double-stranded DNA binding (Figures 4C–E and Supplementary Table 1). These data suggest that E30 causes severe pathological changes in the mouse brain, and specific molecules should be analyzed to elucidate the mechanism of E30 pathogenesis.

**TABLE 3:** Summary of E30-infected mice brain tissue sequencing data quality.

Sample	Raw reads	Clean_reads	Error_rate	Q20	Q30	GC_pct
E30-brain	43,752,896	423,921,50	0.02	98.04	93.44	49.68
E30-brain	45,733,738	44,330,972	0.02	98.16	93.46	49.08
E30-brain	50,430,126	48,242,498	0.02	98.15	93.77	48.47
Mock-1	47,742,674	46,594,900	0.02	98.12	92.96	48.66
Mock-2	45,793,204	44,328,840	0.02	98.14	94.65	48.13
Mock-3	47,472,704	46,486,258	0.02	97.99	94.61	48.34

### Visualization and real-time-PCR validation of differentially expressed genes

**FIGURE 5:**
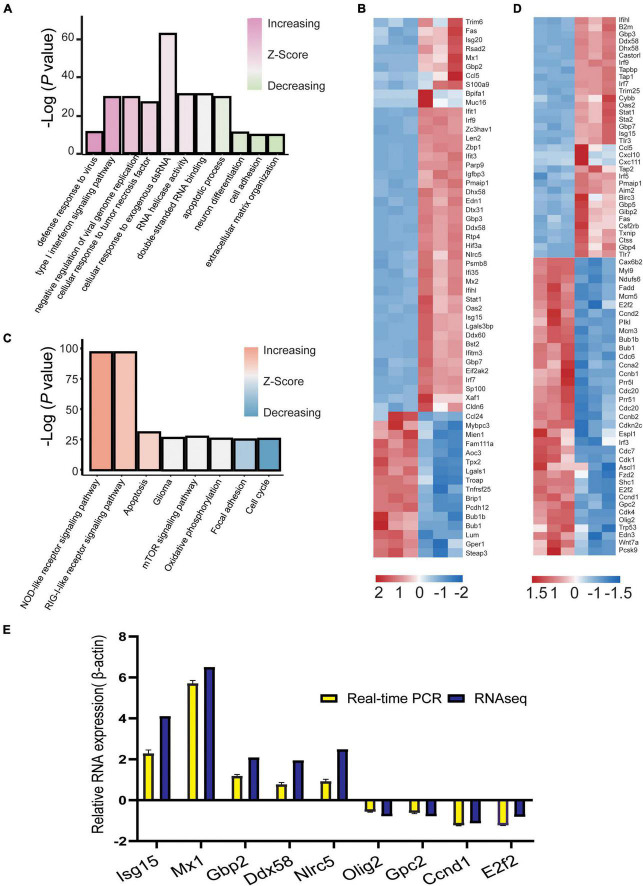
Visualization of differentially expressed genes (DEGs) in echovirus 30-infected suckling mice brain tissue. **(A,C)** GO bar plot analysis of DEGs enriched in GO terms in the brain tissue and **(B,D)** visualization of DEGs enriched in the brain tissue using KEGG. **(E)** The log_2_ fold change of differentially expressed genes in the RNA-Seq data and validation of differentially expressed genes *via* RT-PCR assays.

A GO bar plot was used to explore the expression of DEGs enriched in GO terms and KEGG pathways; genes enriched in viral defense, viral replication, IFN signaling, and NOD-like and RIG-I-like receptor pathways were significantly upregulated. However, DEGs enriched in neuronal differentiation, cell adhesion, extracellular matrix organization, glioma, focal adhesion, and cell cycle were significantly downregulated (Figures 5A,C). We visualized these genes and identified 60 DEGs in GO terms and 58 DEGs in the KEGG pathway. During E30 infection, genes associated with the type I IFN signaling pathway (*Isg15*, *Mx1*, *Mx2*, *Sp100*, *Ifit1*, *Ifit3*, *Ifih1*, *Irf7*, *Irf9*, guanylate-binding proteins 2 [*Gbp2*], and *Stat1*) and defense response to viruses (*Ddx58*, *Ddx60*, *Zbp1*, *Oas2*, *Nlrc5*, and *Eif2ak2*) were significantly upregulated. Genes associated with the neuronal differentiation pathway (*Pcsk9*, *Runx1*, *Cebpb*, *Fzd4*, *Wnt7a*, *Ascl1*, *Fzd2*, *Edn3*, *Olig2*, and *Gpc2*), gliomas (*Shc1*, *Cdk4*, *E2f2*, and *Ccnd1*), and cell cycle (Bub1b, Bub, Ccnb1, Ccnb2, and Cdc20) were significantly downregulated (Figures 5B,D). Nine DEGs were randomly selected from these visualized genes for real-time (RT)-PCR validation. Relative expression levels of these nine genes were determined by normalizing the housekeeping gene (β-actin) transcript data by the 2^–ΔΔCt^ method. These results confirmed that the expression of *Mx1*, *Isg15*, *Nlrc5*, *Gbp2*, and *Ddx58* was upregulated with a relative 1.94–6.5-fold change, while the expression of *Olig2*, *Gpc2*, *E2f2*, and *Ccnd1* was downregulated with a relative 0.78–1.3-fold change, compared to the control group (raw data for Ct values of qPCR results are shown in Supplementary Table 2). Moreover, RNA-Seq analysis data showed a 0.78–5.7-fold increase in log_2_ fold change for *Mx1*, *Isg15*, *Nlrc5*, *Gbp2*, and *Ddx58*, and 0.56–1.23-fold decrease for *Olig2*, *Gpc2*, *E2f2*, and *Ccnd1*. These results suggest that RT-PCR validation is consistent with changes in RNA-seq expression profiles (Figure 5E). In conclusion, the DEGs of E30-infected U251 and mouse brain cells complemented each other to elucidate the molecular-level changes during E30 infection.

## Discussion

There is a growing interest in researchers regarding E30 as a cause of neurological diseases (Maruo et al., 2019; Farshadpour and Taherkhani, 2021; Tian et al., 2021). Hence, this study analyzes the transcriptome sequencing of E30 infection in human glioma (U251) and mouse brain tissue. Previous studies on the neurological symptoms caused by enterovirus have reported that EV-A71 can infect SHSY5Y cells and result in the expression of differential genes in the pathways of nervous system development (Hu et al., 2020). The results of this study indicate that after E30 infected U251 cells, DEGs are enriched in negative regulation of neurogenesis, neurological system process, and pathways of neural crest cell migration. Furthermore, we found that GABRD, GABRB3, GABRE, and GABRQ, which play important roles in neuron cell growth and migration (Niu et al., 2020), are significantly downregulated after infection.

The most enriched pathway for the expression of DEGs *in vitro* was found to be consistent with that observed *in vivo*. In addition, the transcriptome sequencing analysis in this study suggests that the immune response of the mouse brain is upregulated in response to E30 infection. Our data analysis showed that (GBP2, GBP3, and GBP7 in the type I IFN signaling pathway were significantly upregulated after E30 infection. GBPs belong to the family of large GTPases that are induced in response to IFNs, and as the member of the IFN-induced guanosine binding family, it resists microbial immunity and cell death in the event of viral infection (Vestal and Jeyaratnam, 2011). Previous studies have shown that GBP1 and GBP3 possess anti-influenza viral activity (Nordmann et al., 2012), killing and transporting antimicrobial peptides to phagolysosomes by oxidation.

The innate immune response is the first line of viral defense *via* the production of IFNs, pro-inflammatory cytokines, and chemokines (Koyama et al., 2008; Ragu et al., 2020). The death of nerve cells is related to the loss of energy due to mitochondrial dysfunction. The upregulation related to the oxidative phosphorylation (OXPHOS) pathway is particularly important for the differentiation of neurons related to the nervous system (Li et al., 2017; Wang et al., 2017; Pang et al., 2021). In addition, mature nerve cells also need the tricarboxylic acid cycle for energy (Fricker et al., 2018). Previous studies have shown that the zika virus can interfere with cell metabolic homeostasis, thereby facilitating the replication of the virus in cells (Ledur et al., 2020). It is worth noting that the transcriptome sequencing data result reveals that the neurons differentiation, glial cells, and OXPHOS-related genes are downregulated, indicating E30 infection may affect cell mitochondria metabolic abnormalities in order to promote the nervous system related to cell apoptosis, and these cells play an important role in the nervous system development.

In this study, we found that many IFN pathway-related genes were significantly upregulated after E30 infection in the brain tissue of suckling mice and that these genes play an important role in the early infection of host cells (Ledur et al., 2020). In addition, our results suggest that E30 infection may induce neuronal apoptosis by oxidative phosphorylation downregulation. The results indicate that E30 causes neural pathogenesis and provides a theoretical basis for understanding its pathogenic mechanism.

## Data availability statement

The datasets presented in this study can be found in online repositories. The names of the repository/repositories and accession number(s) can be found in the article/Supplementary material.

## Ethics statement

This animal study was reviewed and approved by the Second Ethics Review Committee of the National Institute for Viral Disease Control and Prevention, Chinese Center for Disease Control and Prevention. Written informed consent was obtained from the owners for the participation of their animals in this study.

## Author contributions

YZ: conceptualization, visualization, supervision, project administration, and funding acquisition. QS: methodology, formal analysis, investigation, and resources. JL: software and data curation. QS, JL, BZ, RW, CW, XL, YL, and YZ: validation. QS and JL: writing—original draft preparation. QS, JL, and YZ: writing—review and editing. All authors read and agreed to the published version of the manuscript.

## Footnotes

1
http://david.ncifcrf.gov

## Supplementary material

Supplementary Table 1 Differentially expressed genes in E30-infected U251 cells and in E30-infected ICR suckling mouse brain tissue.

Supplementary Table 2 Raw data for Ct values of qPCR for validation of DEGs.
